# Identification of shared oxidative stress related hub genes in NAFLD and atherosclerosis using bioinformatics and machine learning

**DOI:** 10.1038/s41598-025-34958-5

**Published:** 2026-01-05

**Authors:** Gong Qing, Bo Peng, Huizhen Peng

**Affiliations:** 1 Department of Gastroenterology, The People’s Hospital of Chongqing Liangping District, Chongqing, China; 2 Department of Anesthesiology, Chongqing Traditional Chinese Medicine Hospital, Chongqing, China; 3 Department of Anesthesiology, Chongqing University Cancer Hospital, Chongqing, China; 4 Department of Anesthesiology, Chongqing Cancer Hospital, Chongqing, China; 5 Chongqing Cancer Institute, Chongqing, China

**Keywords:** Non-alcoholic fatty liver disease, Bioinformatics, Oxidative stress, Machine learning, Atherosclerosis, Machine learning, Atherosclerosis, Non-alcoholic fatty liver disease

## Abstract

Non-alcoholic fatty liver disease (NAFLD) is a chronic hepatic illness linked with metabolic dysfunction that is distinguished by aberrant lipid accumulation in hepatocytes. Emerging evidence indicates an elevated risk of atherosclerosis (AS) in NAFLD patients, but the fundamental biological processes are not well comprehended. The present research aims to uncover co-expressed oxidative stress-related hub genes as well as potential mechanisms shared between NAFLD and AS through bioinformatics and machine learning approaches. Gene expression profiles of NAFLD (GSE89632, Allard JP et al.) and AS (GSE100927, Steenman M et al.) were retrieved from the Gene Expression Omnibus (GEO) database. Oxidative stress-related genes were extracted from the GeneCards database. Differential expression analysis identified shared differentially expressed genes between both diseases. Functional enrichment analysis elucidated associated biological processes. Machine learning approaches were employed to screen for oxidative stress-related hub genes. Differential expression analysis revealed 20 co-expressed oxidative stress-related genes (8 upregulated, 12 downregulated) in NAFLD and AS. Functional enrichment analysis revealed that these genes are predominantly associated with biological processes, including the *MAPK* as well as the *FoxO* signaling pathway. Machine learning methods identified *ADM* and *IL2RB* as co-expressed oxidative stress-related hub genes with significant diagnostic potential, with Area Under Curve (AUC) values of 0.787 and 0.887 for AS, and 0.979 and 0.931 for NAFLD. The nomogram model utilizing the two hub genes demonstrated excellent predictive accuracy, with AUC values exceeding 0.9 in both diseases. The present study investigated potential important oxidative stress-related molecules as well as pathogenic processes among NAFLD and AS, offering fresh insights for finding potential biomarkers and treating both diseases in future studies.

**Supplementary Information:**

The online version contains supplementary material available at 10.1038/s41598-025-34958-5.

## Introduction

Non-alcoholic fatty liver disease (NAFLD) is a common chronic liver disease that causes a high level of fat buildup in liver cells (hepatic steatosis ≥ 5%). It is linked to diabetes, obesity, insulin resistance, and metabolic stress-related liver injuries^1,2^. With the global trend of obesity as well as its associated metabolic syndrome, NAFLD has emerged as a primary contributor to chronic liver disease in developed nations, including those in Europe and America, as well as in economically advanced areas of China^3^. According to relevant research data, the global prevalence of NAFLD is as high as 30%, with a higher prevalence in males than females, and an even higher proportion among obese and type 2 diabetes populations^4,5^. In recent years, epidemiological studies have shown that NAFLD patients have a significantly higher risk of atherosclerosis (AS) compared to the general population, as both diseases share various pathophysiological mechanisms linked to metabolic disorders, such as oxidative stress and chronic inflammation^6,7^. However, there is currently a lack of systematic analysis regarding the key molecules and regulatory networks involved in the synergistic progression of these two diseases, particularly in terms of the co-expression patterns of oxidative stress-related genes as well as their potential value in diagnosis, which really needs more in-depth exploration.

Oxidative stress, as a common triggering factor for both NAFLD and AS, can induce lipid peroxidation and mitochondrial dysfunction. This occurs through pathways caused by reactive oxygen species (ROS), leading to the invasion of inflammatory cells, promoting liver fibrosis as well as the creation of arterial plaques^8,9^. Although relevant studies have described the functions of individual oxidative stress genes in NAFLD or AS, such as superoxide dismutase 2 (*SOD2*) as well as NADPH oxidase 4 (*NOX4*), there’s still a gap in identifying the hub genes that these two diseases share and their interactive regulatory networks^10–12^. Furthermore, traditional single-omics analyses often struggle to effectively distinguish disease-specific pathways from common pathways, while a multidimensional screening strategy that combines bioinformatics and machine learning can significantly improve the identification of key molecular targets.

**Fig. 1:**
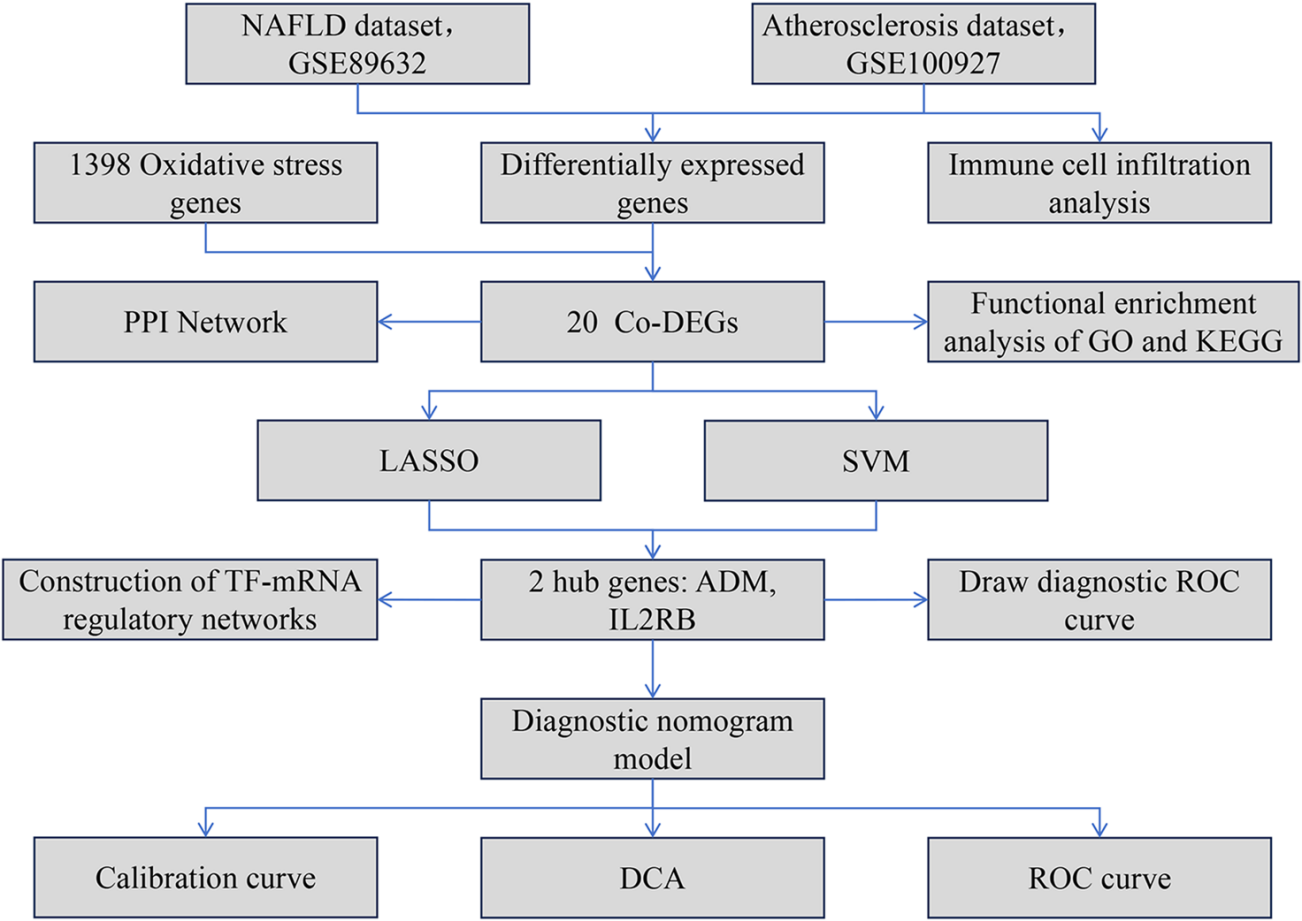
The simplified analysis workflow of this research. The abbreviations in Fig. 1 are as follows: Non-alcoholic fatty liver disease (NAFLD), Atherosclerosis (AS), Common Differentially Expressed Genes (Co-DEGs), Protein–Protein Interaction (PPI), Gene Ontology (GO), Kyoto Encyclopedia of Genes and Genomes (KEGG), Least Absolute Shrinkage and Selection Operator (LASSO), Support Vector Machine (SVM), Receiver Operating Characteristic (ROC), Decision Curve Analysis (DCA).

Based on the above background, this study systematically screened for differentially expressed oxidative stress-related genes co-expressed in both NAFLD as well as AS by integrating transcriptomic data from both diseases, and further identified core hub genes using machine learning approaches (such as Least Absolute Shrinkage and Selection Operator Regression as well as support vector machine analysis). The diagnostic model constructed ultimately aims to reveal novel biomarkers for the comorbidity of NAFLD and AS, offering a theoretical foundation for developing therapeutic targets that cross diseases and promoting research progress in related fields. Figure 1 illustrates the streamlined analytical method employed in this research.

## Materials and methods

### Origin of Raw data

We retrieved and downloaded RNA-seq datasets for non-alcoholic fatty liver disease (NAFLD) as well as atherosclerosis (AS) from the Gene Expression Omnibus (GEO, https://www.ncbi.nlm.nih.gov/geo/). The selection of datasets was primarily based on two criteria: exclusively human-tissue-originated samples, with a minimum of 20 samples per group, to ensure analytical and statistical reliability. The dataset pertaining to NAFLD, identified as GSE89632 (Allard JP et al.), encompasses samples collected from 39 individuals diagnosed with this condition, alongside 24 samples from healthy controls. Additionally, the atherosclerosis dataset (AS), designated as GSE100927 (Steenman M et al.), comprises specimens from 69 individuals suffering from peripheral artery atherosclerosis, in conjunction with 35 control tissue samples. A comprehensive list of 1398 genes associated with oxidative stress, each exhibiting a relevance score of 7 or higher, was retrieved by searching the GeneCards database (https://www.genecards.org/) for additional research, as detailed in Supplementary Table 1. Furthermore, the Transcriptional Regulatory Relationships Unraveled by Sentence-based Text mining‌ (TRRUST) database (https://www.grnpedia.org/trrust/) was employed to forecast transcription factors which exhibit a regulatory relationship with the specified genes of target.

### Screening of differentially expressed genes

First, we downloaded the gene chip data of NAFLD and AS human tissues from the GEO database. Then, we normalized the data using the normalizbetweararray function from the R package “limma”. Subsequently, we performed differential expression analysis using the “limma” package in R, identifying significantly differentially expressed genes (DEGs) with a threshold of |Log2FC|>0.58 and p.adj < 0.05^13^. Subsequently, a volcano plot was created using the “ggplot2” package in R to visualize the DEGs^14^. The “VennDiagram” package in R was utilized to obtain the common differentially expressed genes (Co-DEGs) among NAFLD and AS^15^. Finally, a heatmap of the Co-DEGs was constructed through the “ComplexHeatmap” package in R^16^.

### Functional enrichment as well as PPI network construction

We performed KEGG as well as GO enrichment analysis with the Co-DEGs from NAFLD and AS utilizing the “clusterProfiler” R package^17–21^. In the search tool for recurring instances of neighbouring genes (STRING) database (https://cn.string-db.org/), protein-protein interaction (PPI) information is obtained by setting a confidence score cutoff of ≥ 0.15. Subsequently, the obtained PPI network is visualized using Cytoscape software.

### Machine learning methods for screening Co-expressed hub genes related to oxidative stress

To further screen for co-expressed hub genes related to oxidative stress in NAFLD and AS, the least absolute shrinkage and selection operator (LASSO) regression method utilized for variable selection to improve prediction accuracy, was first employed using the “glmnet” packag^22^. Next, the “e1071” package was utilized for support vector machine (SVM) analysis of the variables to obtain model prediction results and visualize them^23^. Finally, the intersection of genes selected by LASSO and SVM was taken to determine the co-expressed hub genes related to oxidative stress in NAFLD and AS.

### Development along with evaluation of the nomogram model

We developed a nomogram model relying on the selected hub genes with the “rms” package and visualized it. The nomogram model’s predictive capacity was then assessed utilizing calibration curves, decision curve analysis (DCA), as well as receiver operating characteristic (ROC) curves.

### Evaluation of Immune-Related cells infiltration

To evaluate the infiltration of immune-related cells in NAFLD as well as AS tissue samples, we calculated the immune cell infiltration in each sample based on the ssGSEA algorithm provided in the R package - GSVA, referencing the markers of 24 types of immune cells provided in previous literature^24,25^.

### Statistical methods

This study’s statistical assessment was conducted by applying the software R (version 4.2.1). With respect to continuous variables, if the data followed a normal distribution, t-tests (in the states of two groups) or one-way analysis of variance (in the states of multiple groups) were used to compare the differences among groups. For categorical variables as well as continuous variables which failed to have a normal distribution, a test called the wilcoxon examination was applied. Each of the statistical analyses were classified as statistically significant at a p-value of less than 0.05.

## Results

### Identification of Co-expressed oxidative Stress-related differential genes

**Fig. 2:**
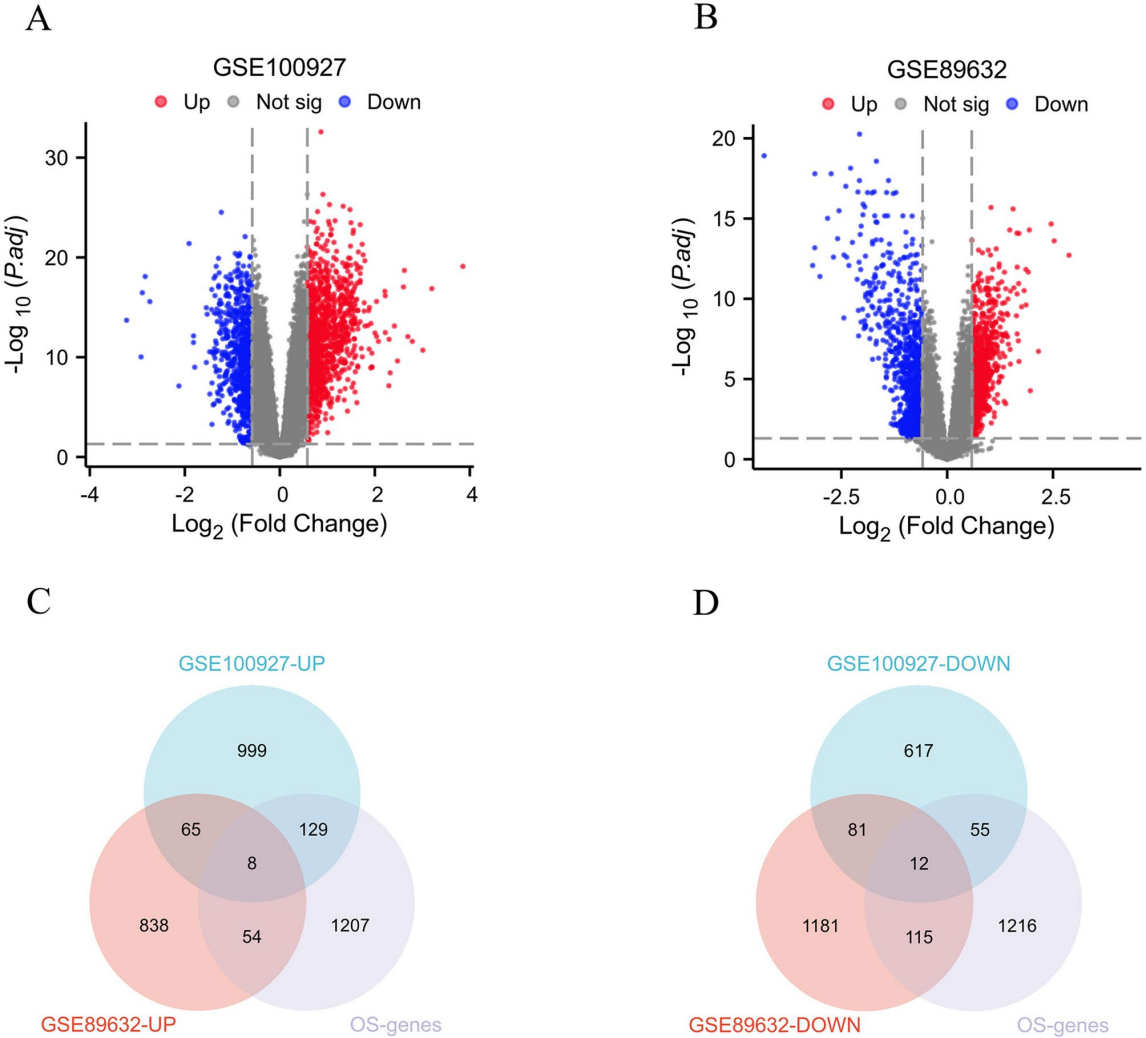
Identification of Co-DEGs between NAFLD and AS. (**A**) Volcano plot of DEGs in AS; (**B**) Volcanic plot of DEGs in NAFLD; (**C**) Venn diagram of upregulated Co-DEGs in AS and NAFLD; (**D**) Venn diagram of downregulated Co-DEGs in AS and NAFLD.

**Fig. 3:**
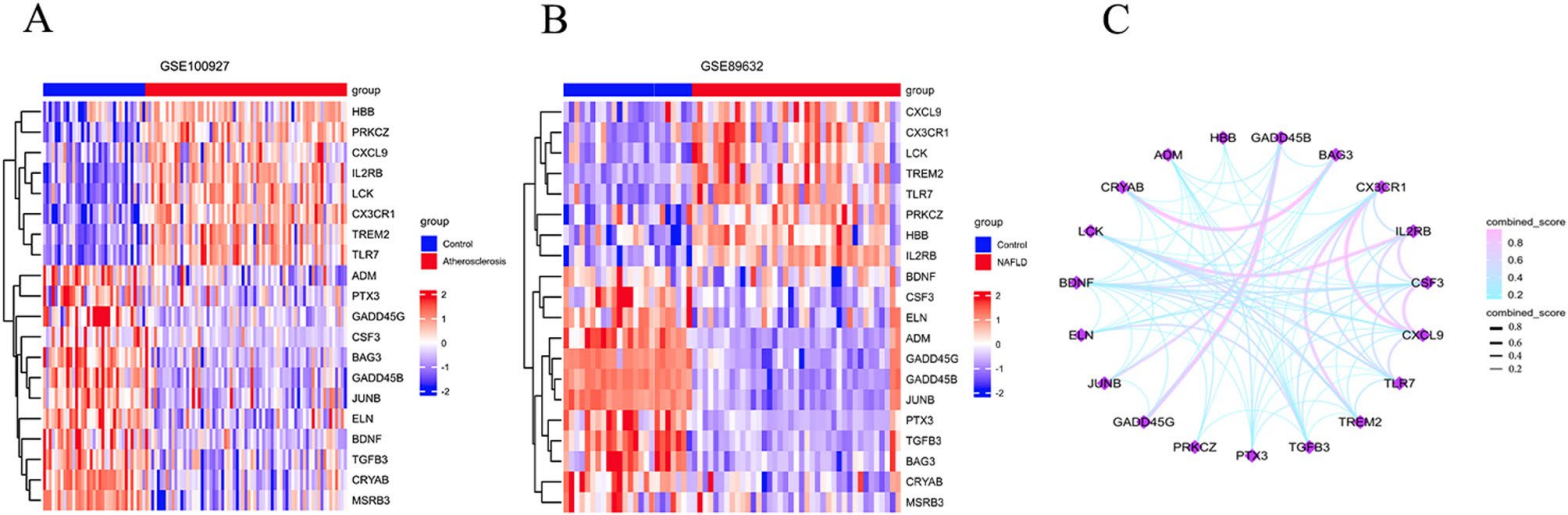
The 20 Co-DEGs between NAFLD and AS. (**A**) Heatmap of Co-DEGs in AS; (**B**) Heatmap of Co-DEGs in NAFLD; (**C**) The PPI network of Co-DEGs.

Based on the differential gene screening criteria (|Log2FC|>0.58 as well as p.adj < 0.05), a total of 1966 genes with differential expression (DEGs) were identified in the atherosclerosis (AS) samples (Fig. 2A), and 2354 DEGs were identified in the non-alcoholic fatty liver disease (NAFLD) samples (Fig. 2B), shown as volcano plots, as detailed in Supplementary Table 2. Subsequently, the DEGs from AS and NAFLD were intersected with the 1398 oxidative stress-related genes, resulting in the identification of 20 common differentially expressed genes (Co-DEGs), including 8 upregulated genes (Fig. 2C) as well as 12 downregulated genes (Fig. 2D), which are presented in a Venn diagram, as detailed in Supplementary Table 3. The relative expression for the 20 Co-DEGs in AS (Fig. 3A) and NAFLD (Fig. 3B) samples were displayed in heatmap form. Finally, a protein interaction network of the 20 Co-DEGs was constructed (Fig. 3C), as detailed in Supplementary Table 4.

### Functional enrichment analysis of Co-expressed differential genes

**Fig. 4:**
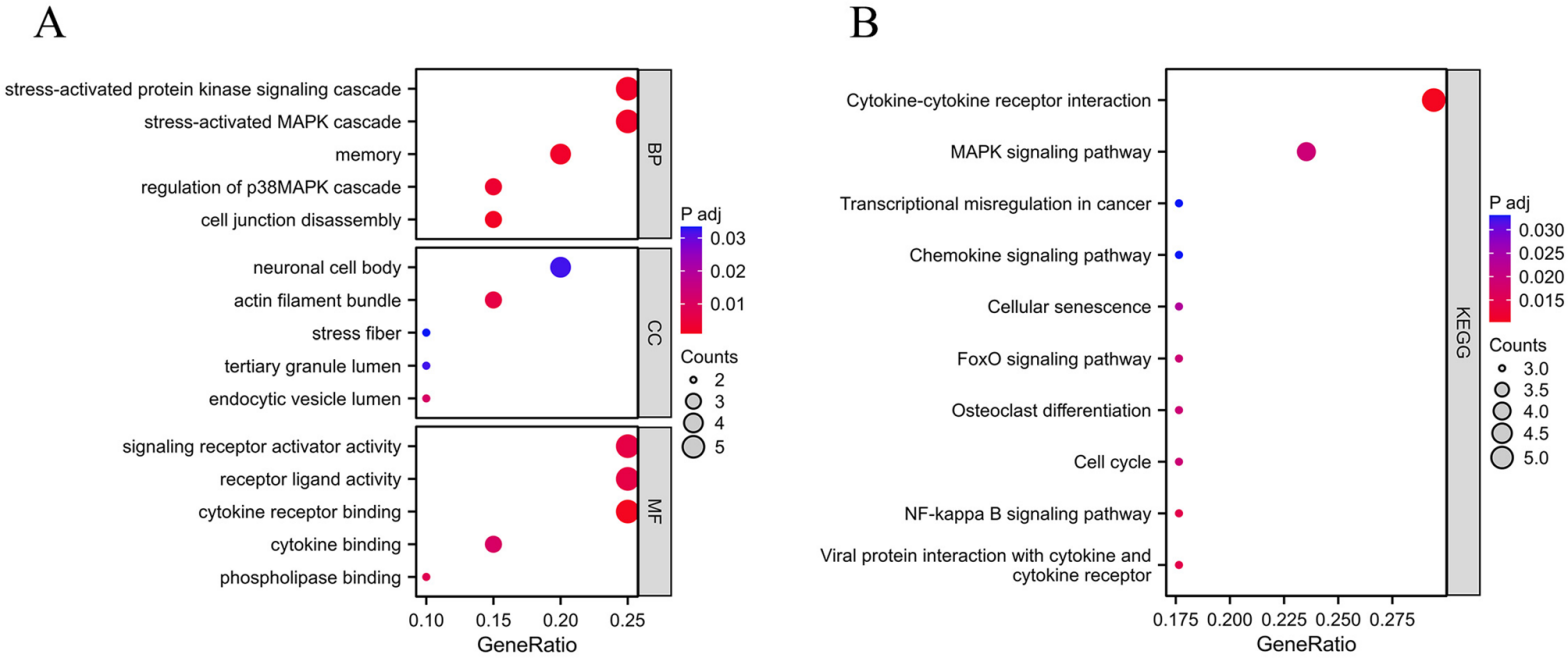
Functional Enrichment Analysis. (**A**) GO enrichment analysis; (**B**) KEGG enrichment analysis.

To further explore the biological roles of the Co-DEGs within NAFLD and AS, KEGG as well as GO enrichment analyses were performed on the selected 20 Co-DEGs. The GO enrichment analysis results revealed that the Co-DEGs were predominantly engaged in biological processes including “stress-activated MAPK cascade”, “signaling receptor activator activity”, “stress-activated protein kinase signaling cascade”, as well as “cytokine receptor binding” (Fig. 4A). The KEGG enrichment analysis results showed that the Co-DEGs predominantly participated in biological processes including “*MAPK* signaling pathway”, “*FoxO* signaling pathway”, “Chemokine signaling pathway”, “Cytokine-cytokine receptor interaction”, as well as “Cellular senescence” (Fig. 4B), as detailed in Supplementary Table 5.

### Screening of Co-expressed oxidative Stress-related hub genes through machine learning techniques

**Fig. 5:**
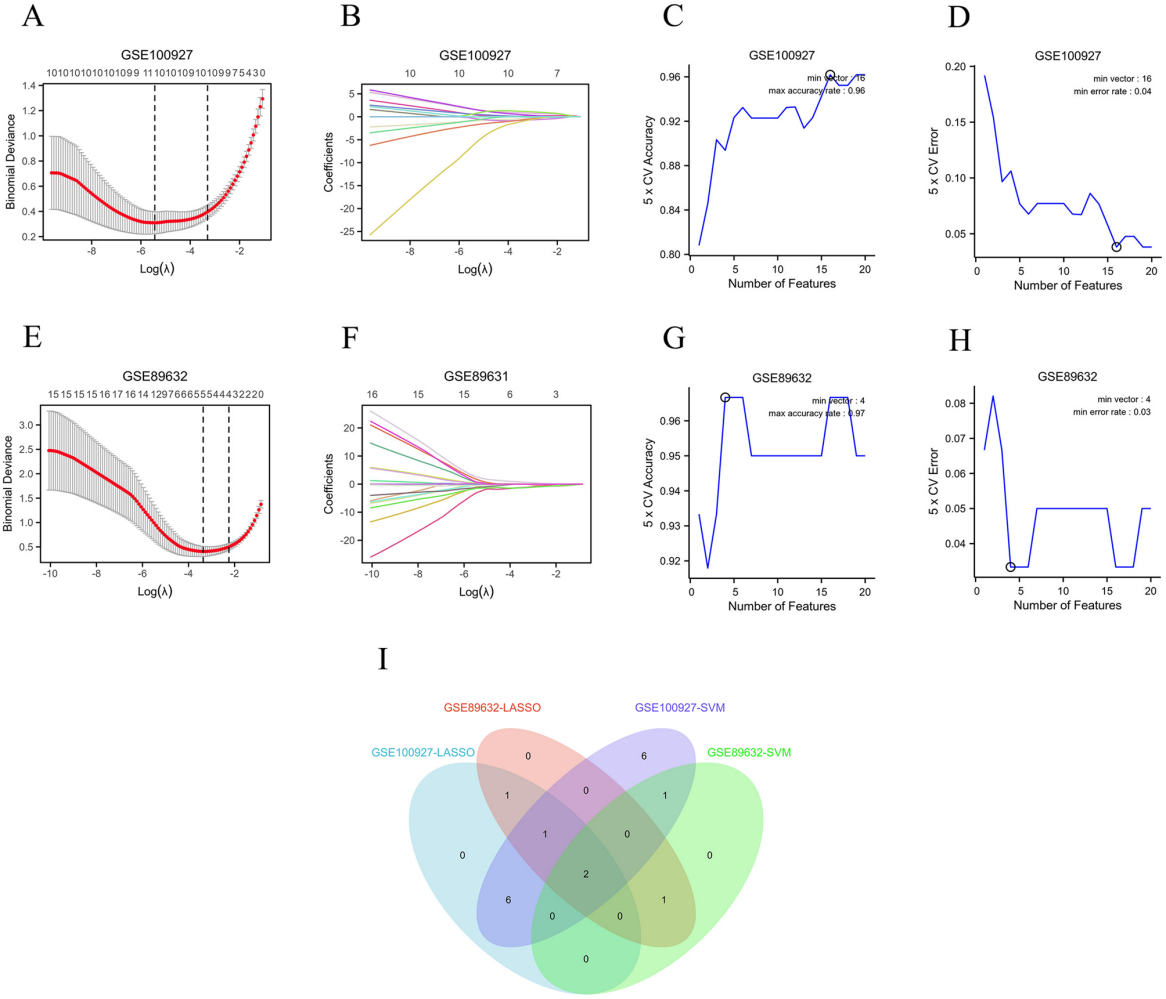
Screening of Co-expressed Oxidative Stress-related Hub Genes Through Machine Learning Techniques. (**A**, **E**) Lasso cross validation diagram in AS and NAFLD; (**B**, **F**) Lasso coefficient analysis diagram in AS and NAFLD; (**C**, **G**) Accuracy of feature variables based on SVM method in AS and NAFLD; (**D**, **H**) Error of feature variables based on SVM method in AS and NAFLD; (**I**) Venn diagram of screening hub genes through machine learning methods.

In order to screen for co-expressed hub genes related to oxidative stress in NAFLD and AS, least absolute shrinkage and selection operator (LASSO) regression analysis was first utilized on the selected 20 Co-DEGs. The λ (lambda.min) value was determined through 10 fold cross validation, and variables with non-zero lambda values were selected as feature variables. The results revealed that 10 feature genes were identified through LASSO regression analysis in the AS dataset (Fig. 5A-B), while 5 feature genes were selected in the NAFLD dataset (Fig. 5E-F), as detailed in Supplementary Table 6. Subsequently, support vector machine (SVM) analysis was performed on the selected 20 Co-DEGs, the SVM model used gaussian radial basis kernel function (kernel="radial”) with parameters set to gamma = 0.125 and C = 2. Which resulted in the identification of 16 feature genes in the AS dataset (Fig. 5C-D) and 4 feature genes in the NAFLD dataset (Fig. 5G-H). Finally, the intersection of the four gene sets selected by LASSO regression analysis and SVM analysis yielded two key co-expressed oxidative stress-related hub genes: *ADM* and *IL2RB* (Fig. 5I).

### Diagnostic value of *ADM* and *IL2RB* in NAFLD and AS and construction of TF-mRNA regulatory network

**Fig. 6:**
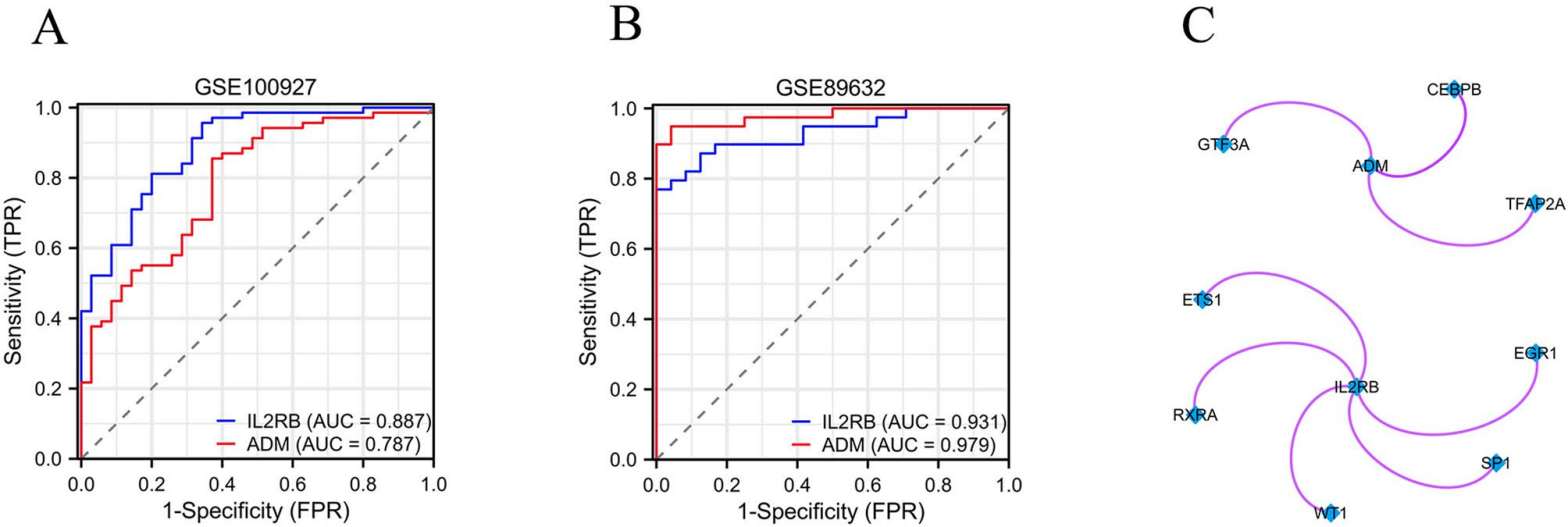
Diagnostic Value of *ADM* and *IL2RB* in NAFLD and AS and Construction of TF-mRNA Regulatory Network. (**A**) The ROC curves of hub gene *ADM* and *IL2RB* in AS; (**B**) The ROC curves of hub gene *ADM* and *IL2RB* in NAFLD; (**C**) TF-mRNA regulatory network.

Receiver operating characteristic (ROC) curves as well as the area under the curve (AUC) were created to assess the diagnostic value of the two hub genes, *ADM* and *IL2RB*, in NAFLD and AS. The findings indicated that the AUC values for *ADM* and *IL2RB* in AS were 0.787 and 0.887, respectively (Fig. 6A), while in NAFLD, the AUC values were 0.979 and 0.931, respectively (Fig. 6B), indicating that the two hub genes identified in this study have good diagnostic value in both NAFLD and AS, as detailed in Supplementary Table 7. The predicted transcription factors for *ADM* and *IL2RB* were acquired according to the TRRUST database. The findings show that *GTF3A*, *CEBPB*, as well as *TFAP2A* are anticipated regulatory transcription factors for *ADM*, while *EGR1*, *ETS1*, *RXRA*, *SP1*, and *WT1* are predicted regulatory transcription factors for *IL2RB* (Fig. 6C).

### Construction of the nomogram model and evaluation of diagnostic value

**Fig. 7:**
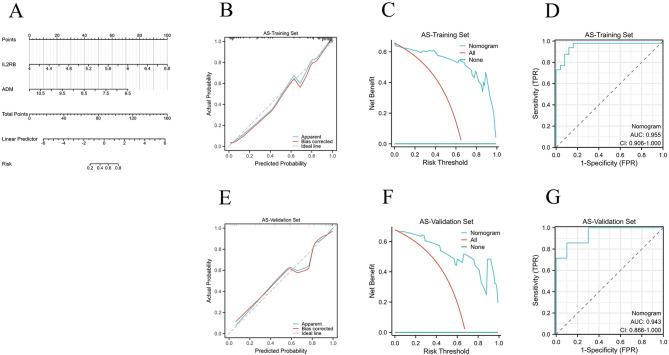
Construction of the Nomogram Model and Evaluation of Diagnostic Value in the AS training set and validation set. (**A**) The nomogram model in the AS training set; (**B**, **E**) The calibration curves of nomogram model in the AS training set and validation set; (**C**, **F**) The DCA curves of nomogram model in the AS training set and validation set; (**D**, **G**) The ROC curves of nomogram model in the AS training set and validation set.

**Fig. 8:**
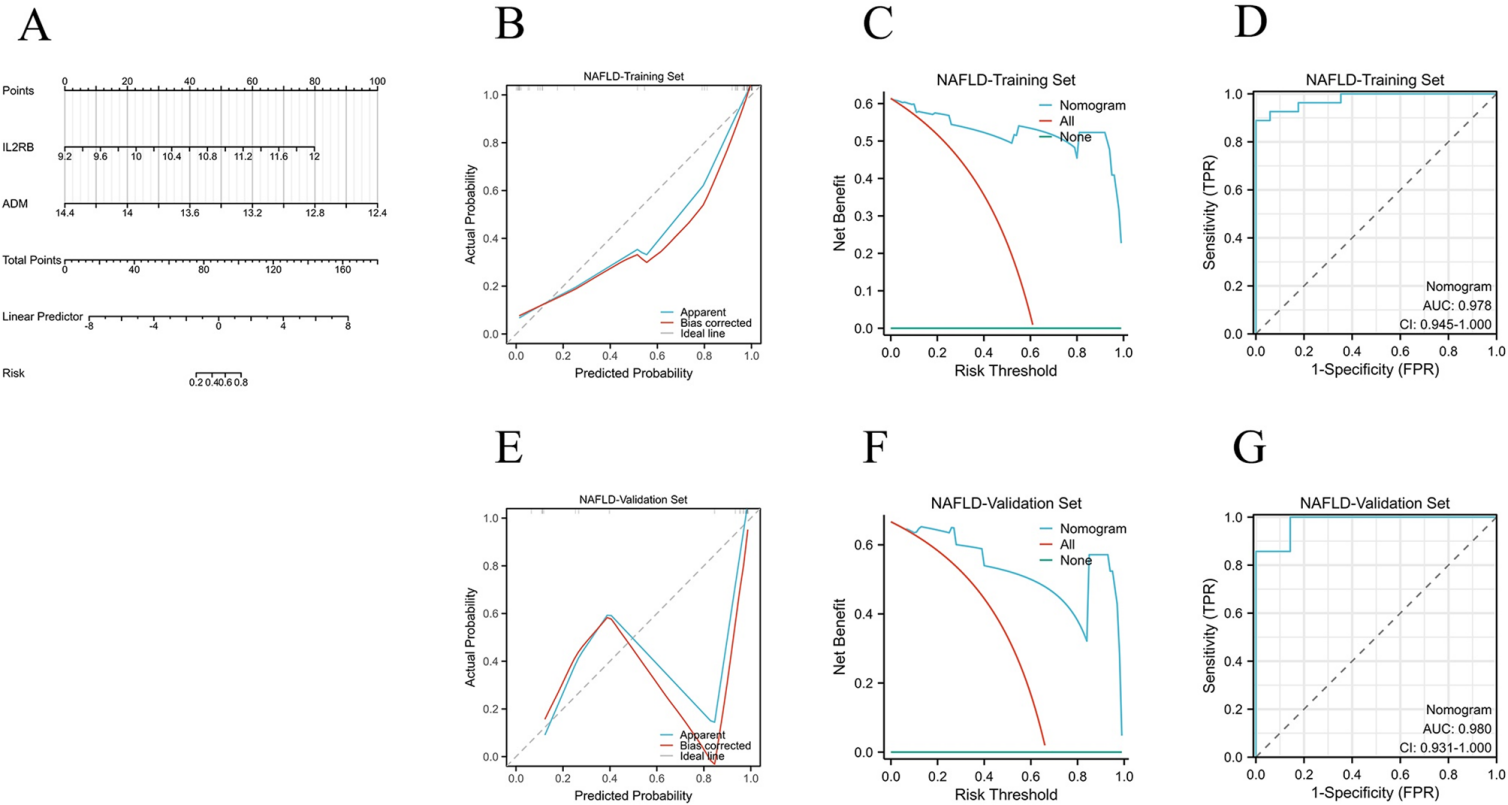
Construction of the Nomogram Model and Evaluation of Diagnostic Value in the NAFLD training set and validation set. (**A**) The nomogram model in the NAFLD training set; (**B**, **E**) The calibration curves of nomogram model in the NAFLD training set and validation set; (**C**, **F**) The DCA curves of nomogram model in the NAFLD training set and validation set; (**D**, **G**) The ROC curves of nomogram model in the NAFLD training set and validation set.

The AS dataset (GSE100927, Steenman M et al.) and the NAFLD dataset (GSE89632, Allard JP et al.) were divided into training and validation sets in a ratio of 7:3, respectively. Based on the selected hub genes *ADM* and *IL2RB*, diagnostic nomogram models were constructed in the training sets of AS and NAFLD (Figs. 7A and 8A). To better evaluate the predictive performance of the nomogram model, In the training and validation sets of AS and NAFLD, calibration curves were used for calibration assessment (Figs. 7B and E and 8B and E), decision analysis curves (DCA curves) were used for clinical applicability assessment (Figs. 7C and F and 8C and F), and ROC curves and AUC values were used for discrimination assessment (Figs. 7D and G and 8D and G). The results demonstrated that the AUC levels of the nomogram model for both AS and NAFLD exceeded 0.9, surpassing the AUC levels of individual genes.

### Outcomes of Immune-Related cells infiltration assessment

**Fig. 9:**
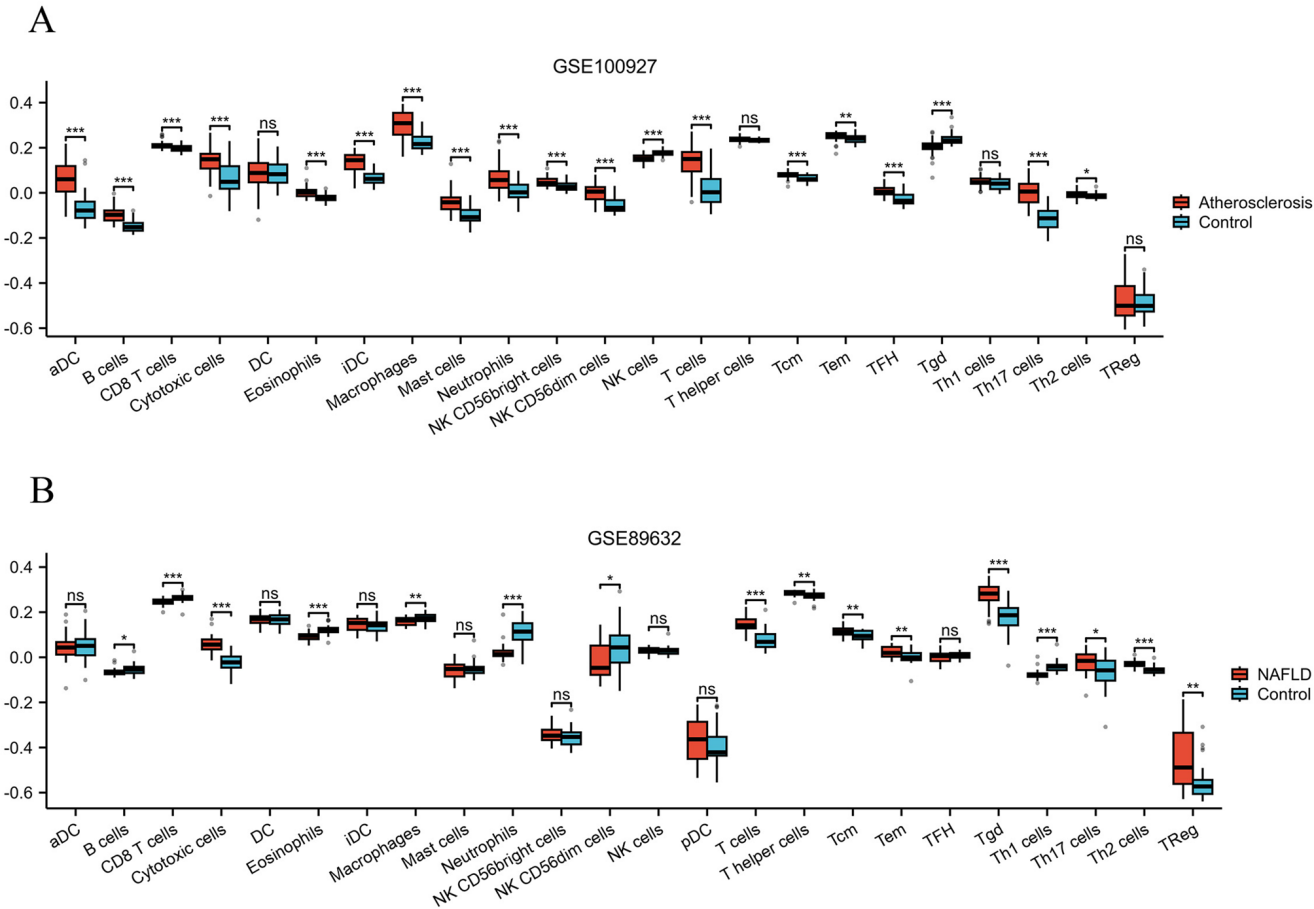
Outcomes of Immune-Related Cells Infiltration Assessment. (**A**) Immune-Related Cells Infiltration in AS; (**B**) Immune-Related Cells Infiltration in NAFLD.

The immune-related cell infiltration analysis showed that compared with the control group, the levels of “aDC”, “B cells”, “CD8 T cells”, “Cytotoxic cells”, “Eosinophils”, “iDC”, “Macrophages”, “Mast cells”, “Neutrophils”, “NK CD56bright cells”, “NK CD56dim cells”, “T cells”, “Tcm”, “Tem”, “TFH”, and “Th17 cells”, “Th2 cells” were significantly increased in AS tissue samples, while the levels of “NK cells” and “Tgd” were significantly decreased (Fig. 9A). In NAFLD tissue samples, the levels of “Cytotoxic cells”, “T cells”, “T helper cells”, “Tcm”, “Tem”, “Tgd”, “Th17 cells”, “Th2 cells”, and “TReg” were significantly increased compared to the control group, while the levels of “B cells”, “CD8 T cells”, “Eosinophils”, “Macrophages”, “Neutrophils”, “NK CD56dim cells”, and “Th1 cells” were significantly decreased (Fig. 9B), detailed information can be found in Supplementary Table 8.

## Discussion

Non-alcoholic fatty liver disease (NAFLD) as well as atherosclerosis (AS) are escalating global health concerns. NAFLD happens when there’s abnormal fat buildup in the liver, with pathological alterations which can advance from simple fatty liver (SFL) towards non-alcoholic steatohepatitis (NASH), possibly eventually leading to liver fibrosis, cirrhosis, and even carcinoma of the hepatocytes^26,27^. Meanwhile, AS is a major factor leading to cardiovascular diseases, characterized by thickening and hardening of arterial walls, which may ultimately result in severe consequences such as heart disease and stroke^28^. The pathogenesis of NAFLD and AS is complex, with intersections in metabolic syndrome, insulin resistance, and significant influence from oxidative stress^29,30^. Therefore, exploring the common mechanisms of these two diseases and their potential clinical applications is of great research significance.

This study utilized bioinformatics methods to comprehensively analyze NAFLD and AS data from the GEO database, successfully recognizing 20 co-expressed differential genes that are primarily involved in multiple key biological functions, such as “*MAPK* signaling pathway”, “*FoxO* signaling pathway”, and so on. Further machine learning methods ultimately identified two key hub genes, *ADM* and *IL2RB*, whose ROC curve analyses indicated that they have high diagnostic value in both NAFLD and AS. The nomogram model constructed based on these two hub genes demonstrated high predictive accuracy, with AUC values greater than 0.9 in both diseases, surpassing the AUC values of individual genes. Additionally, the dysregulation of the immune microenvironment has a potential association with the progression of these two diseases, indicating that oxidative stress may significantly contribute to the pathogenic mechanisms of NAFLD and AS. These findings not only provide new biomarkers for clinical diagnosis but also offer potential targets for future therapeutic strategies.

Based on the co-expressed differential genes identified in this study, we found that they are mainly enriched in several key biological processes such as the “*MAPK* signaling pathway” and “*FoxO* signaling pathway” during functional enrichment analysis, suggesting that these pathways and their related genes may play an important role in the common pathological processes of NAFLD and AS. Previous studies have shown that the *MAPK* signaling pathway (containing *JNK*, *ERK*, *p38* subfamilies) regulates cell proliferation, apoptosis, inflammation, as well as oxidative stress through phosphorylation cascades^31^. In NAFLD, studies have found that lipotoxicity (such as free fatty acids and cholesterol crystals) promotes ROS generation by activating *JNK* and *p38 MAPK* in hepatocytes, leading to mitochondrial dysfunction and ultimately causing hepatocyte apoptosis^32^. Another study showed that *MAPK* activation can induce the secretion of pro-inflammatory cytokines, for instance *IL-6* as well as *TNF-α*, exacerbating liver inflammation and fibrosis^33^. In AS, previous studies have found that oxidized low-density lipoprotein (ox-LDL) accelerates plaque formation by activating the endothelial cell *MAPK* pathway, promoting monocyte adhesion and macrophage foam cell formation^34^. In addition, the persistent activation of *JNK* is also associated with the phenotypic transition of vascular smooth muscle cells (VSMCs), leading to plaque instability^35^. Previous studies have shown that *FoxO* transcription factors (such as *FoxO1*, *FoxO3*) are key regulators of antioxidant enzymes (*SOD*, *CAT*), and their activity is inhibited by the insulin/*AKT* pathway^36^. Research has found that in NAFLD, insulin resistance leads to decreased *AKT* activity and increased nuclear translocation of *FoxO1*, which activates lipolysis-related genes (such as *ATGL*), exacerbating liver lipotoxicity^37^. A study in AS found that ox-LDL activates pro-apoptotic genes mediated by *FoxO1* (such as Bim) by inhibiting endothelial cell *AKT* activity, leading to endothelial barrier disruption^38^. Additionally, the absence of *FoxO3* can exacerbate vascular ROS accumulation and the risk of plaque rupture^39^. In NAFLD and AS, the chemokine signaling pathway plays a central role by mediating the chemotaxis of immune cells and the inflammation response^40^. A study in NAFLD found that lipid accumulation activates Kupffer cells, releasing chemokines such as *CCL2* and *CCL5*, which recruit monocytes and neutrophils to infiltrate the liver, driving the progression of steatosis to non-alcoholic steatohepatitis (NASH) and fibrosis^41^. Another previous study in AS found that endothelial injury induces the expression of *CX3CL1* and *CCL2*, mediating the migration of monocytes to the subendothelial space through receptors (such as *CCR2*), differentiating into foam cells and enhancing plaque inflammation and instability^42^. Although this study suggests that signaling pathways such as *MAPK* and *FoxO* may play important roles in the comorbidity mechanism of NAFLD and AS through bioinformatics analysis, their specific mechanisms of action still need to be further validated through subsequent experiments.

In this study, we successfully identified *ADM* and *IL2RB* as co-expressed oxidative stress-related hub genes between NAFLD and AS. These two genes play important roles in various biological processes, particularly in regulating immune responses and metabolic homeostasis. *ADM* is a multifunctional peptide hormone with vasodilatory, anti-inflammatory, antioxidant, and metabolic regulatory effects^43^. *ADM* mitigates liver inflammation by stimulating the *PI3K*/*Akt* signaling pathway and inhibiting the secretion of pro-inflammatory substances, for instance *TNF-α* as well as *IL-6*^44^. In AS, *ADM* promotes nitric oxide (NO) release and improves vascular dilation by activating the *AKT*/eNOS pathway in endothelial cells^45^. *IL2RB* is a common receptor subunit for *IL-2* and *IL-15*, primarily affecting immune-related metabolism and inflammatory responses. In the progression of NAFLD, imbalance in *IL-2RB* signaling may lead to dysregulation of the hepatic immune microenvironment, exacerbating inflammatory responses and fibrosis^46^. In AS, *IL-2RB* may be involved in the activation of macrophages as well as T cells within plaques, facilitating the secretion of pro-inflammatory cytokines (for instance *IFN-γ*), thereby accelerating plaque progression^47^. Identifying these critical genes enhances our comprehension of the disease mechanisms behind NAFLD and AS, while also presenting novel potential targets for future treatment interventions.

In the analysis of immune cell infiltration, this study found that various immune cell infiltration levels were significantly elevated in AS tissues, and NAFLD samples also showed changes in immune cell composition, especially in the variations of macrophages, T cells, and B cell subsets. Existing studies have confirmed that macrophage polarization plays an important role in the formation and progression of atherosclerotic plaques^48,49^. In addition, multiple studies have shown that the differentiation of T cells and the various inflammatory factors they secrete are key driving factors for plaque instability and vascular injury^50,51^. Furthermore, previous studies on NAFLD have found that the remodeling of immune cells in the liver environment is an important factor promoting lipid accumulation, hepatocyte injury, and fibrosis progression^52,53^. This study not only deepens the understanding of the immunopathological mechanisms of NAFLD and AS but also provides a new theoretical basis for therapeutic strategies targeting immune cells and their molecular signals.

This study provides important insights into the potential molecular mechanisms linking NAFLD and AS, but there are still several important limitations that need to be addressed to ensure an objective and comprehensive interpretation of the research results. This study primarily analyzes publicly available datasets based on the GEO, and the sample size may be limited and derived from specific populations or geographic areas, which may restrict the generalizability of the findings to all patient subgroups. Future research that includes a broader and more diverse cohort will help enhance the representativeness of the results and strengthen the robustness of the observations. Bioinformatics research heavily relies on existing computational algorithms and knowledge repositories. Although these tools are powerful and widely used, they also have limitations. First, the analysis results may be sensitive to the specific parameter thresholds and normalization methods chosen. Second, these methods inherently depend on the currently known biological knowledge and relationships stored in public databases. Therefore, novel, less-explored, or newly discovered mechanisms may not be effectively captured by our analysis, and the biological significance of identified biomarkers and pathways, although reasonable based on existing literature, may be overinterpreted in the absence of experimental validation. In future research, we will strive to further validate the potential biomarkers and biological significance discovered in this study through experiments.

## Conclusion

In summary, the present research successfully identified key oxidative stress-related genes within NAFLD and AS through systematic bioinformatics analysis and constructed effective predictive models. In particular, *ADM* and *IL2RB* serve as potential biomarkers, providing novel avenues for early diagnosis and targeted treatment of these diseases.

## Supplementary Information

Below is the link to the electronic supplementary material.

Supplementary Material 1

Supplementary Material 2

Supplementary Material 3

Supplementary Material 4

Supplementary Material 5

Supplementary Material 6

Supplementary Material 7

Supplementary Material 8

Supplementary Material 9

## Data Availability

The original data presented in the study are publiclyavailable. This data can be found here from the Gene Expression Omnibus (GEO) database: https://www.ncbi.nlm.nih.gov/geo accession numbers (GSE89632, Allard JP et al.) and (GSE100927, Steenman M et al.).
