# Supplementary figures and images for: Identification of shared oxidative stress related hub genes in NAFLD and atherosclerosis using bioinformatics and machine learning
Source: Sci Rep. 2026 Jan 5;16:4644. doi: 10.1038/s41598-025-34958-5 (PMC12868795; doi:10.1038/s41598-025-34958-5)

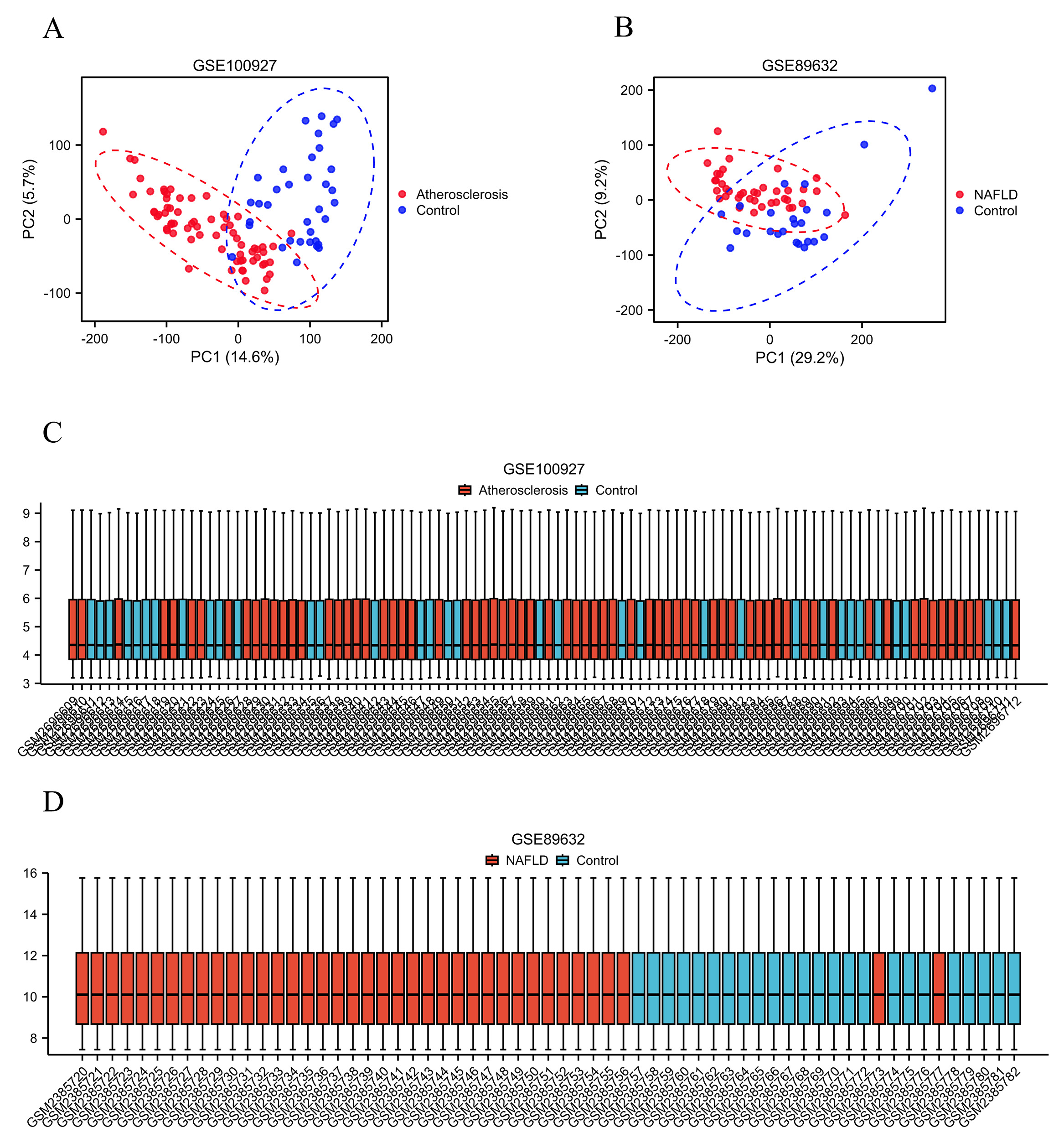

Supplement: Supplementary file 9 — Supplementary Material 9 [file 41598_2025_34958_MOESM9_ESM.jpg]
